# Examining the Nutritional Quality of Canadian Packaged Foods and Beverages with and without Nutrition Claims

**DOI:** 10.3390/nu10070832

**Published:** 2018-06-27

**Authors:** Beatriz Franco-Arellano, Marie-Ève Labonté, Jodi T. Bernstein, Mary R. L’Abbé

**Affiliations:** 1 Department of Nutritional Sciences, Faculty of Medicine, University of Toronto, Toronto, ON M5S 3E2, Canada; beatriz.francoarellano@mail.utoronto.ca (B.F.-A.); Marie-Eve.Labonte@fsaa.ulaval.ca (M.-È.L.); jodi.bernstein@mail.utoronto.ca (J.T.B.); 2 Institute of Nutrition and Functional Foods, Laval University, Québec, QC G1V 0A6, Canada

**Keywords:** nutritional quality, nutrition claims, nutrient profiling, Canada, foods and beverages, food label

## Abstract

Nutrient content claims, health claims, and front-of-pack symbols (henceforth referred to as “nutrition claims” in the present study) are often found on food labels in Canada. However, it is currently unknown whether foods and beverages (F&Bs) carrying nutrition claims have a more favourable nutritional profile than those without such claims. This study examined differences in the global nutritional quality, as determined by the Food Standards Australia New Zealand Nutrient Profiling Scoring Criterion (FSANZ-NPSC), of Canadian F&B bearing nutrition claims as compared to those without, as well as in their nutritional composition. Data (*n* = 15,184) was obtained from the University of Toronto 2013 Food Label Information Program. Forty-two percent of F&Bs carrying nutrition claims (*n* = 2930/6990) were found to be ineligible to carry claims based on the FSANZ-NPSC, in comparison to 66% of F&Bs without (*n* = 5401/8194, *p* < 0.001). Sugars and sweets, and miscellaneous products were the food categories with larger proportions of foods carrying nutrition claims not meeting the FSANZ-NPSC eligibility criteria. F&Bs with nutrition claims had fewer calories, less saturated fat, sodium, and sugar, and higher content of protein and fibre than comparable products without nutrition claims (*p* < 0.05 in all cases). In conclusion, nearly half of F&Bs carrying nutrition claims in Canada did not meet the FSANZ-NPSC threshold, although Canadian products carrying nutrition claims have an overall “healthier” profile than their counterparts without such claims.

## 1. Introduction

Nutrient content claims, health claims, and front-of-pack symbols (henceforth referred to as “nutrition claims” in the current study) are often found on food labels [1,2,3,4,5,6,7]. Nutrition claims are described by the Codex Alimentarius (CODEX) as “any representation which states, suggests or implies that a food has particular nutritional properties including, but not limited to, the energy value and to the content of protein, fat and carbohydrates, as well as the content of vitamins and minerals” [8]. Many countries have adopted CODEX recommendations and regulated the use of nutrition claims. However, many of these nutrition claims are often displayed on the labels of foods and beverages (F&Bs) of lower nutritional quality [3,9,10,11]. Research has shown that F&Bs with nutrition claims may mislead consumers by highlighting certain beneficial nutrients or components (e.g., fibre), while minimizing information on nutrients of public health concern (e.g., sodium, sugar, and saturated fat) [12,13].

Nutrition claims have also been found to have an impact on consumers’ choices [14]. For example, a recent meta-analysis found that F&Bs carrying nutrition claims are 75% more likely to be chosen than an identical F&Bs without such claims [14]. Nutrition claims also appear to increase the “halo effect”, which refers to the consumer tendency, in the presence of a nutrition claim on a label, to give a higher nutritional “rating” to other attributes not highlighted in the claim itself [15]. This effect increases the consumers’ perceptions of the nutritional quality of less healthy F&Bs [16,17,18] and/or increases consumers’ willingness to buy F&Bs with claims as compared to F&Bs with no claims [18]. Thus, public health authorities and health-focused non-governmental organizations are advocating limited use of nutrition claims, particularly in F&Bs with high contents of sodium, sugar, and saturated fat [19].

In Canada, mandatory nutrition labelling has been in place since 2003 under the Food and Drugs Act [20,21], with the primary objective to protect consumers against being misled. These regulations made compulsory for manufacturers to provide a Nutrition Facts table (NFt) and a list of ingredients on most packaged food products (except for a few products such as baked in-store products, coffee, vinegar, and spices, among others). These regulations also provided the requirements for the use of voluntary nutrition claims [20,21]. As such, in order to display nutrition claims products must meet certain nutrient thresholds and use prescribed wording, and accurate information should be provided about composition in relation to the nutrition claim being made (see Supplementary Table S1 for detailed information on each type of claim included in the present study). Regulated nutrition claims include nutrient content claims and health claims. Nutrient content claims (NCCs) are those that “describe the amount of a nutrient in a food”, and some examples include “excellent source of calcium” or “low in sodium” [1]. Although nutrient content claims are the type of claim most often used on food labels worldwide as well as in Canada [2,3,4,7,9,11,22], products bearing such claims are not always indicative of higher nutritional quality [3,6,11]. Health claims are “any representation in labelling or advertising that states, suggests, or implies that a relationship exists between consumption of a food or an ingredient in the food and a person’s health” [21,23], and comprises two subtypes: disease risk reduction claims (which are statements that link a food or constituent of a food to reducing the risk of developing a diet-related disease or condition; for example, “a healthy diet rich in a variety of vegetables and fruit may help reduce the risk of some types of cancer”) and nutrient-function claims (which describe the well-established roles of energy or nutrients that are essential for the maintenance of good health or for normal growth and development; for example, “This product is a source of calcium. Calcium helps build strong bones and teeth”) [21,23]. Although a number of disease risk reduction claims are allowed by Health Canada [24], less than 2% of labels display such claims [4].

Other general health claims (considered in the present study as “unregulated” nutrition claims) broadly representing “symbols, logos and specific words (e.g., healthy for you, etc.)” are often presented on the front-of-pack (FOP) of labels [25]. FOP symbols have been defined as “systems that use nutrient criteria and symbols to indicate that a product has certain nutritional characteristics. Symbols are often placed on the principal display panel of the product, but may also be found on the side, top, or back panels or on shelf-tags” [26]. The use of FOP symbols has also increased, with at least 20% of products in Canada carrying FOP symbols on their labels [4]. However, FOP symbols, particularly those presented as ‘health logos’ or those showing only information without interpretation (e.g., Guideline Daily Amounts (GDA), Facts Up Front), might mislead consumers [27,28,29,30]. Studies have also found that consumers perceived products with FOP symbols as more healthful and lower in negative nutrients, and these symbols failed to help consumers discriminate healthier from less healthy food choices [27,28,29,30]. For instance, Emrich and colleagues conducted a mock-package experiment with over 3000 Canadians testing consumer responses to different FOP symbols on a frozen meal. In the absence of a Nutrition Facts table, consumers perceived products with FOP symbols as of higher nutritional quality [27]. Another randomized controlled trial conducted in the United States showed that participants that had the ‘Facts Up Front’ as a FOP symbol had a misconception of the nutritional quality of packaged foods that were shown to them, with participants underestimating amounts of nutrients to limit, while overrating products with nutrients to encourage [28]. In addition, other studies have found that, in general, products with FOP symbols do not have higher nutritional quality [30,31]. Currently, FOP symbols are not specifically regulated by the Canadian government and consequently many unique FOP symbols are found on Canadian food labels [32]. As such, the Government of Canada has identified the need to incorporate FOP labelling into its regulations, and recently proposed amendments to the latter with the objective of providing consumers with “clear and consistent front-of-package information on key nutrients of concern” [33].

Previous studies have examined the prevalence of nutrition claims on F&Bs labels in Canada [4,32,34]. However, since information on the overall nutritional quality of products is not compulsory for products carrying nutrition claims, it is unknown whether F&Bs with nutrition claims are of higher nutritional quality than those without such claims. The current work examined differences in the global nutritional quality, as determined by the Food Standards Australia New Zealand Nutrient Profiling Scoring Criterion (FSANZ-NPSC), and the specific nutritional composition of Canadian products bearing nutrition claims in comparison to those without.

## 2. Materials and Methods

### 2.1. Study Design

This study was a cross-sectional analysis of the University of Toronto Food Label Information Program (FLIP), version 2013.

Briefly, the FLIP is a database that contains information on packaged foods and non-alcoholic beverages, representing approximately 75% of the Canadian grocery retail market shares, as described in detail elsewhere [35]. FLIP 2013 (*n* = 15,342) was collected by systematically scanning grocery store shelves from the four leading retail chains and by taking photographs of all products, using a smartphone application [35]. All packaged foods and beverages with a mandatory Nutrition Facts table (NFt) were collected, including all flavour variations and national and private labels. Baby and toddler foods, natural health products (e.g., herbal remedies), or seasonal products (e.g., Easter chocolates, Christmas eggnog) were excluded from data collection, although meal replacements were collected. Label data were uploaded onto an online database platform specially designed for this purpose. Data captured included nutrition information, ingredients list, price, brand, container size, and universal product code (UPC). Nutrition information was captured in the “as purchased” form. For products requiring preparation (e.g., canned soups, muffin mix), “as consumed” data were also determined using the ESHA Food Processor software and food composition data from the 2013 Canadian Nutrient File (CNF) [36]. Trained staff classified products into 22 food categories (as defined in Schedule M of the Food and Drug Regulations [37], version in force at the time of the data collection) and an additional category for meal replacements. All forms of regulated nutrient content claims and health claims were classified using the Food and Drug Regulations [21], and unregulated front-of-pack symbols were identified on all food labels by the research team using a decision tree developed for this purpose, as described elsewhere [4].

One hundred and eleven products originally collected in the FLIP 2013 database were excluded from analyses for the present study if they were natural health products (*n* = 1), had declared caloric values >20% from caloric values determined by Atwater calculations (*n* = 55), or were meal replacements (*n* = 55). An additional 47 products were also excluded from analyses in this study because of the lack of declaration for a nutrient required to assess global nutritional quality using the FSANZ-NPSC (method detailed in the section below). The final number of products analysed in this study was therefore *n* = 15,184.

### 2.2. Assessment of the Nutritional Quality of Foods Using a Nutrient Profiling System

#### 2.2.1. Justification for the Use of the FSANZ-NPSC as a Method for Assessing Nutritional Quality

The World Health Organization (WHO, Geneva, Switzerland) has defined nutrient profiling (NP) as “the science to evaluate the nutritional quality of food and beverages in a systematic method, that could allow for transparency and fair comparison among those foods and beverages” [38]. Several nutrient profiling models have been developed to assist health authorities advancing policies, such as the regulation of nutrition claims [39]. The Food Standards Australia New Zealand Nutrient Profiling Scoring Criterion (FSANZ-NPSC) was used to determine the nutritional quality of all F&Bs in the database [19]. This nutrient profiling system was chosen because it was specifically developed to determine the eligibility of a food or beverage to carry health claims [19].

#### 2.2.2. Applying the FSANZ-NPSC to the FLIP Database

Foods and beverages in FLIP 2013 were initially classified into one of three possible categories defined by the FSANZ-NPSC: beverages (Category 1), any food item not in category 1 or 3 (Category 2), and cheese with a high calcium content (>320 mg/100 g) and fats (e.g., oil, butter) (Category 3). Nutrient information (energy, saturated fat, sugars, sodium, protein, and fibre) was extracted from the NFt displayed on labels. The content of fruit, vegetables, nuts, and legumes (FVNL), a key component of the FSANZ-NPSC, was also determined. Given the lack of quantitative ingredient declarations in Canada, a method to estimate the FVNL content was developed by our group using the ingredient list; the detailed method is described by Bernstein et al. [40]. Once nutrient information was standardized per 100 g or 100 mL in the “as purchased” (i.e., “not as consumed”) form, points for each individual nutrient and FVNL content were determined according to the FSANZ-NPSC. “As purchased” information was used in order to maximize inter-category comparability given that preparation instructions can vary from brand to brand [5], and also to be able to compare our results with similar studies [5,7,9,10]. The nutritional quality, in the form of an overall score, was calculated per product by adding points for nutrients to limit (e.g., sodium) and deducting points for nutrients or components to encourage (e.g., fibre), respectively, according to the following formula [19,41]: FSANZ-NPSC Score = Energy + Saturated Fat + Sugars + Sodium − Protein − Fibre − FVNL. Foods and beverages were classified as eligible to carry a claim (i.e., “healthier”), using established cut-off scores in the FSANZ-NPSC: <1 for beverages, <28 cheese with calcium content >320 mg/100 g and fats (e.g., oil, butter), and <4 for the remaining foods [19,41]. The F&Bs that did not meet their respective cut-offs were classified as not eligible to carry a claim (i.e., “less healthy”).

### 2.3. Statistical Analyses

Products carrying nutrition claims (i.e., nutrient content claims, health claims (specifically the disease risk reduction claims subtype; structure function claims were not assessed in the current study), and front-of-pack symbols) that had already been classified in FLIP 2013 as part of a previous study [4] were included. Products without such claims were also included in the analyses. Total number of products with and without claims analysed was *n* = 15,184. 

The overall proportion of F&Bs not meeting the FSANZ-NPSC threshold was calculated for products with nutrition claims, comprising nutrient content claims (including subtypes of nutrient content claims; e.g., fat claims, sodium claims), disease risk reduction claims, and front-of-pack symbols (including subtypes of front-of-pack symbols; e.g., nutrient-specific symbols, summary indicator symbols), as well as for all products without claims. The Chi-squared test was used to evaluate differences in the proportion of F&Bs with and without claims not meeting the FSANZ-NPSC threshold. The proportion of F&Bs with these claims was also determined by the Schedule M food category [37]. The Chi-squared test was also used to determine if the proportion of F&Bs not meeting the FSANZ-NPSC threshold was statistically different for those with and without nutrition claims (*p* < 0.05) in each food category.

Means and standard deviations (SDs) for energy, saturated fat, sodium, sugar, protein, and fibre were determined for F&Bs with and without each of the different types of nutrition claims (i.e., nutrition claims, nutrient content claims, disease risk reduction claims and FOP symbols) in all foods products analysed (total *n* = 15,184). Student’s *T*-test or Mann–Whitney *U* test (for those nutrients that were not normally distributed) were used to evaluate differences in nutrient content between products with and without each type of nutrition claims. A comparison of the nutrient content between F&Bs with and without each type of nutrition claims was also conducted in each of the food categories comprising a substantial proportion of claims (i.e., >40% of products in the category carrying claims). Analyses were conducted in R Studio (https://www.r-project.org).

## 3. Results

### 3.1. Analyis of Foods and Beverages with and without Nutrition Claims that Would Not Be Eligible to Carry Claims (as Determined by the FSANZ-NPSC)

#### 3.1.1. Overall Nutritional Quality of Products in FLIP 2013

Analyses showed that 55% of products in the database (*n* = 8331/15,184) would not be considered eligible to carry claims (i.e., they did not meet FSANZ-NPSC threshold).

#### 3.1.2. Analysis of the Nutritional Quality of Foods in FLIP 2013, by Type of Claim

**Figure 1:**
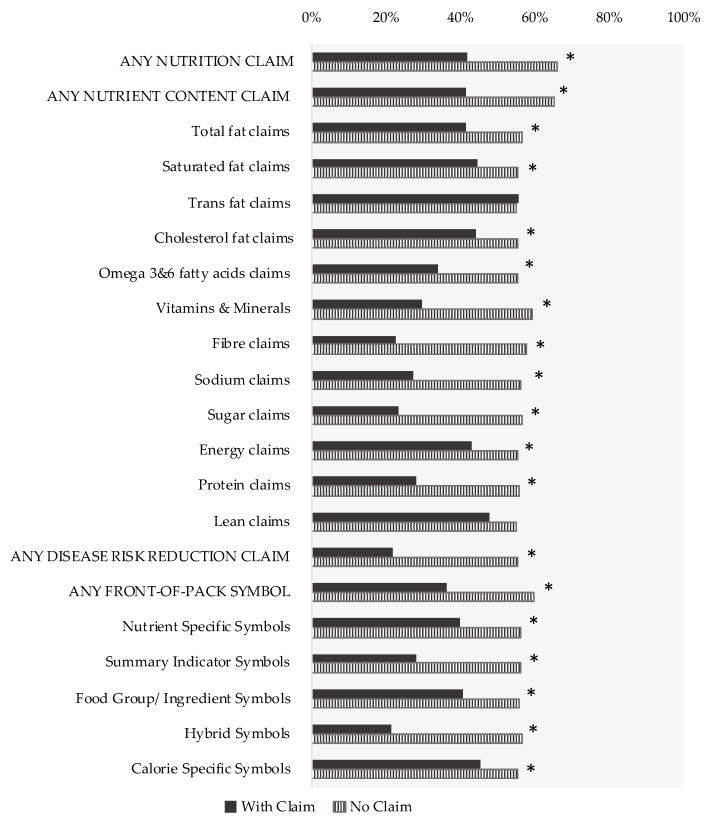
Overall proportion of foods and beverages with and without nutrition claims that would not be eligible to carry claims, as determined by the Food Standards Australia New Zealand Nutrient Profiling Scoring Criterion (FSANZ-NPSC), per type of claim (*n* = 15,184). A product was considered not eligible to carry claims if it did not meet its corresponding threshold of the FSANZ-NPSC. Values per 100 g/mL “as purchased” were used to determine the score. Nutrition claims include any nutrient content claims, health claims and/or front-of-pack symbols (Supplementary Tables S1 and S2 for details). A product can carry more than one type of claim and the addition of the proportion of nutrition claims can exceed 100%. * Denotes statistically significant difference based on χ^2^ test (*p* < 0.05).

Forty-six percent of products included in this study carried nutrition claims (*n* = 6990/15,184) whereas 54% of products did not carry claims (*n* = 8194/15,184). Almost 42% (*n* = 2930/6990) of products carrying claims were considered “less healthy” (i.e., ineligible to carry claims according to FSANZ-NPSC), in comparison to 66% (*n* = 5401/8194) of foods without claims (*p* < 0.001). (Figure 1; detailed results in Supplementary Table S2).

The proportion of products not meeting the FSANZ-NPSC was lower in products carrying nutrient content claims than in products without such claims (41%, *n* = 2687/6501 vs. 65%, *n* = 5644/8683, respectively, *p* < 0.001) (Figure 1). Detailed analyses also revealed similar results for most subtypes of nutrient content claims, except for *trans*-fat claims and lean claims, for which the proportion of F&Bs not meeting the FSANZ-NPSC threshold did not significantly differ between products with and without these specific claims (both *p* ≥ 0.29).

As previously reported, disease risk reduction claims were much less prevalent on food labels, with only 1.5% (*n* = 226/15,184) of products carrying these types of claims [4]. However, 22% (*n* = 49/226, *p* < 0.001) of products carrying disease risk reduction claims would not be eligible to carry claims using the FSANZ-NPSC, in comparison to 55% of those without (*n* = 8282/14,958) (Figure 1; detailed results in Supplementary Table S2).

The proportion of products not meeting the FSANZ-NPSC was also lower in products with unregulated FOP symbols than in their counterparts without FOP symbols (36%, *n* = 1110/3056 vs. 59%, *n* = 7221/12,128, respectively, *p* < 0.001). Analyses at the subtype level (e.g., by type of front-of-pack symbol) revealed similar results (Figure 1; detailed results in Supplementary Table S2).

#### 3.1.3. Analysis of the Nutritional Quality of Foods in FLIP 2013, by Food Category

Analyses by food category also showed that on one hand, certain food categories such as eggs and eggs substitutes and legumes had very low proportions of foods with nutrition claims not meeting the eligibility criteria (0% and 1%, respectively); however, these food categories only represent a small proportion of all F&Bs in FLIP 2013 (0.4%, *n* = 56/15,184 and 1.2% *n* = 180/15,184, respectively) (Table 1).

On the other hand, food categories with a large prevalence of F&Bs in FLIP 2013, such as bakery products (14%, *n* = 2083/15,184) and sauces, dips and gravies (8.1%, *n* = 1223/15,184) had much larger proportions of foods carrying nutrition claims not meeting the FSANZ-NPSC eligibility criteria (60%, *n* = 605/1004 and 55.5%, *n* = 132/238, respectively; Table 1). In addition, other categories such as sugars and sweets (84.4%, *n* = 124/147), miscellaneous foods (81%, *n* = 111/137), dessert toppings and fillings (70%, *n* = 14/20), fats and oils (68.6%, *n* = 190/277), snacks (68.6%, *n* = 328/478) and desserts (59.6%, *n* = 229/384) had more than half of their products carrying claims that were considered as not eligible to carry those according to the FSANZ-NPSC. These food categories also represent approximately 50% of products in the database. It is worth noting that almost 29% of fruit and fruit juices with nutrition claims were not eligible to carry such claims (*n* = 214/746), (see Table 1).

Analyses per type of nutrition claim (i.e., nutrient content claims, disease risk reduction claims, FOP symbols) showed that legumes, and eggs and eggs substitutes were also commonly the food categories with fewer F&Bs that were not eligible to carry claims (see Table 2, Table 3 and Table 4).

### 3.2. Nutritional Composition of Foods and Beverages with and without Nutrition Claims

F&Bs with nutrition claims had fewer calories (*p* < 0.001) and less saturated fat (*p* < 0.001), sodium (*p* < 0.001), and sugar (*p* < 0.001), and a higher content of protein (*p* = 0.042) and fibre (*p* < 0.001) than F&Bs without nutrition claims (Table 5, see Table S3 for category level data). Observations were similar across all types of claims, although calorie content did not differ between F&Bs with disease risk reduction claims and those without (*p* = 0.95). Also, protein content was lower in F&Bs with disease risk reduction claims (*p* = 0.003) and in F&Bs with FOP symbols as compared with F&Bs not carrying such claims (both *p* < 0.001).

## 4. Discussion

This is the first comprehensive study, to our knowledge, to investigate the nutritional quality of foods with and without different types of nutrition claims in Canada. This study also identified the proportion of foods and beverages carrying different types of nutrition claims per food category that would not be eligible to carry claims, based on the FSANZ-NPSC. 

As previously reported [4], almost half of F&Bs in the Canadian food supply carried at least one nutrition claim (either nutrient content claim, disease risk reduction claim or front-of-pack symbol). However, results from this study showed that 42% of these would be considered not eligible to carry claims based on the FSANZ-NPSC. This is concerning considering the influence that nutrition claims have on consumers’ choices [14]. Results from this study align with findings from other studies that have demonstrated that products with nutrition claims do not always have a more favourable nutritional profile as compared to similar F&Bs without claims [31,42]. The present study is also in line with other research which suggests that nutrition claims are mostly used for food marketing [7,42,43,44], particularly when claims are used on “less healthy” F&Bs [3,34]. Interestingly, this research also found that the overall proportion of F&Bs considered eligible to carry claims (45%) was similar to studies conducted in Australia and New Zealand (45%) [5] and another study involving five European countries (43%) [10], suggesting that the nutritional quality of F&Bs in industrialized countries might be comparable. However, it could be more important to evaluate what have been the outcomes of policies limiting the use of nutrition claims that have been implemented in those countries [45], which could serve as a precedent if similar policies are contemplated in Canada. 

There is currently global interest of governments in adapting FOP systems rather than developing new ones; however, it is critical to evaluate if the particular characteristics underlying a model fit with the public health policy in appraisal [46]. This study highlighted that F&Bs with FOP symbols overall have a higher proportion of “healthier” products than their counterparts without FOP symbols, as determined by the FSANZ-NPSC. For instance, more than 70% of foods carrying hybrid symbols or summary indicator systems, 60% of F&Bs with FOP symbols emphasizing food groups or particular ingredients, and 55% of F&Bs carrying calorie specific symbols were considered “healthier”. However, given there are currently several FOP systems used on labels, having one simple FOP system, as proposed by the Government of Canada [33], could support consumers towards choosing healthier foods by consistently highlighting key nutrition information [33] (e.g., nutrients of public health concern). The introduction of government-endorsed FOP symbols might eventually lead to a “healthier” food supply and an increase in product reformulation [47]. For instance, an analysis conducted in Australia that looked at the nutritional composition of F&Bs before and after the adoption of a voluntary but standardized FOP labelling scheme showed F&Bs were being reformulated towards a “healthier” profile after the introduction of such labelling scheme [48]. A similar pattern was observed in the Chilean food supply after a mandatory FOP system was adopted in the country, where up to 20% of F&Bs have been reformulated [49].

Although results from this research revealed that a significant proportion of products carrying nutrition claims did not meet the FSANZ-NPSC threshold for carrying a claim (41% of F&Bs carrying nutrient content claims, 21% of F&Bs carrying disease risk reduction claims, and 36% of F&Bs front-of-pack symbols), the overall nutritional quality of F&Bs with nutrition claims still was considered “healthier” compared to F&Bs without claims. These results also indicated that the nutritional quality of products is food category dependent. For instance, at least 70% of products with nutrition claims in certain food categories such as sugars and sweets, miscellaneous foods, and desserts toppings and fillings would not be eligible to carry claims. In other categories like fats and oils, snacks, bakery products, desserts, sauces, dips, and gravies, half of products with claims were not considered eligible to bear such claims. Although these food categories tend to be limited in dietary guidelines, they carry a substantial number of claims. Thus, from a public health perspective, preventing “less healthy” food categories from carrying nutrition claims could encourage the promotion of healthier options and reformulation among manufacturers [47,48,49].

Consumers tend to evaluate the nutritional quality of F&Bs based on single nutrients (such as those conveyed by nutrition claims) instead of assessing the nutritional properties of F&Bs as a whole [50]. The results of this study highlight the need for policymakers to consider the implications of allowing the use of nutrition claims on “less healthy” F&Bs, the potential for overall nutritional quality as a criterion for a F&Bs to be eligible to carry such claims, and the role that a nutrient profiling model can have in identifying such products. For instance, a finding of this study was that the nutritional quality did not differ between F&Bs with and without *trans*-fat claims, which is the third most common claim in Canada [4]. The Government of Canada is already proposing a modification to the Food and Drug Regulations that will allow updates to the regulation of nutrition claims more efficiently [33]. Data presented in this study provide a comprehensive evaluation of the use of nutrition claims in Canada. 

This study has a few limitations. First, the classification of nutrition claims differs globally, which restricts comparisons between countries. For instance, other studies in Europe and in New Zealand have also used the FSANZ-NPSC [5] to evaluate the nutritional quality of products with claims; however, since the latter were classified differently, comparisons to our study can be done only at an overall level, and not by type of claims. Future investigations could address this limitation by classifying claims using international definitions, such as those proposed by the International Network for Food and Obesity/non-communicable disease Research, Monitoring and Action Support (INFORMAS) [12], which are based on CODEX food standards for the use of nutrition claims on labels [8]. Second, the selection and use of one nutrient profiling model inherently excludes the use of others. However, the FSANZ-NPSC has been endorsed by the Australian and New Zealand Governments, specifically to limit the use of health claims on products which do not meet certain nutritional criteria [19,51]. The FSANZ-NPSC was developed based on another highly-validated model (United Kingdom Ofcom model), and has also been applied by other researchers to assess the nutritional quality of F&Bs with claims [3,9,10,52]. Third, the data were collected in 2013, which does not acknowledge that some products could have been reformulated in recent years or that product packages could have been updated to display more or fewer claims. However, FLIP 2013 was still the most recent and largest database available on branded food packages in Canada at the time this study was conducted. Lastly, the use of data in the “as purchased” form, although it allows for comparisons to similar studies [5,7,9,10], could potentially have restricted some products to be eligible to carry claims under the FSANZ-NPSC.

Strengths of this study include the large number of products included in these analyses. Other studies have only evaluated subsamples of the food supply [3,9,10] or certain food categories [40,52,53]. This is also the first study, to our knowledge, that has investigated the nutritional quality of foods with and without different types of nutrition claims in Canada using a nutrient profiling system specifically developed to assess the eligibility of food products to carry claims. A similar earlier Canadian study only investigated the nutritional composition (i.e., content of specific nutrients) of products carrying front-of-pack symbols [31].

## 5. Conclusions

Canadian food and beverages carrying nutrition claims on their labels have an overall “healthier” profile than foods and beverages which do not carry those claims. Proportions of F&Bs eligible to carry claims varied by type of nutrition claim and by food category. Still, many products that would not be considered eligible to carry claims based on the FSANZ-NPSC carried such claims, potentially misleading consumers to perceive these products as more nutritious options. This research highlights current practices in the use of nutrition claims on Canadian packaged F&Bs, particularly in foods and beverages with poorer nutritional profiles. This study also draws attention to the importance of considering the overall nutritional quality of products as a criterion for carrying nutrition claims, and the relevance of using nutrient profiling systems to identify and limit “less healthy” food products from carrying nutrition claims. The data presented here could inform policymakers and could help to track changes in the nutritional quality of the food supply over time, in light of the proposed updates to the labelling regulations in Canada.

## Figures and Tables

**Table 1:** Proportion of products carrying nutrition claims that would or would not be eligible to carry such claims according to the FSANZ-NPSC, by Schedule M food category (*n* = 15,184).

Schedule M Food Category	All Products		Nutrition Claims (Present)					Nutrition Claims (Absent)				
	*n*	*%*	*n*	*Eligible*		*Not Eligible*		*n*	*Eligible*		*Not Eligible*	
				*n*	*%*	*n*	*%*		*n*	*%*	*n*	*%*
Bakery Products	2083	13.7%	1004	399	39.7%	605	60.3%	1079	175	16.2%	904	83.8%
Beverages	481	3.2%	231	148	64.1%	83	35.9%	250	69	27.6%	181	72.4%
Cereals and Other Grain Products	981	6.5%	568	430	75.7%	138	24.3%	413	368	89.1%	45	10.9%
Dairy Products and Substitutes	1237	8.1%	791	537	67.9%	254	32.1%	446	134	30.0%	312	70.0%
Desserts	827	5.4%	384	155	40.4%	229	59.6%	443	38	8.6%	405	91.4%
Dessert Toppings and Fillings	115	0.8%	20	6	30.0%	14	70.0%	95	6	6.3%	89	93.7%
Egg and Egg Substitutes	56	0.4%	30	30	100.0%	0	0.0%	26	23	88.5%	3	11.5%
Fats and Oils	535	3.5%	277	87	31.4%	190	68.6%	258	104	40.3%	154	59.7%
Marine and Fresh Water Animals	440	2.9%	209	168	80.4%	41	19.6%	231	172	74.5%	59	25.5%
Fruit and Fruit Juices	1088	7.2%	746	532	71.3%	214	28.7%	342	202	59.1%	140	40.9%
Legumes	180	1.2%	98	97	99.0%	1	1.0%	82	82	100.0%	0	0.0%
Meat, Poultry, Their Products and Substitutes	895	5.9%	312	159	51.0%	153	49.0%	583	90	15.4%	493	84.6%
Miscellaneous category	449	3.0%	137	26	19.0%	111	81.0%	312	48	15.4%	264	84.6%
Combination Dishes	1348	8.9%	514	386	75.1%	128	24.9%	834	425	51.0%	409	49.0%
Nuts and Seeds	220	1.4%	116	82	70.7%	34	29.3%	104	85	81.7%	19	18.3%
Potatoes, Sweet Potatoes and Yams	140	0.9%	75	68	90.7%	7	9.3%	65	34	52.3%	31	47.7%
Salads	70	0.5%	27	24	88.9%	3	11.1%	43	29	67.4%	14	32.6%
Sauces, Dips, Gravies and Condiments	1223	8.1%	238	106	44.5%	132	55.5%	985	261	26.5%	724	73.5%
Snacks	794	5.2%	478	150	31.4%	328	68.6%	316	74	23.4%	242	76.6%
Soups	455	3.0%	262	167	63.7%	95	36.3%	193	64	33.2%	129	66.8%
Sugars and Sweets	739	4.9%	147	23	15.6%	124	84.4%	592	4	0.7%	588	99.3%
Vegetables	828	5.5%	326	280	85.9%	46	14.1%	502	306	61.0%	196	39.0%
Total	15,184	100.0%	6990	4060	58.1%	2930	41.9%	8194	2793	34.1%	5401	65.9%

A product was considered not eligible to carry claims if it did not meet its corresponding threshold of the Food Standards Australia New Zealand Nutrient Profiling Scoring Criterion (FSANZ-NPSC). Values per 100 g/mL “as purchased” were used to determine the score. A product (i.e., food or beverage) can carry more than one type of claim and thus the addition of individual proportions of nutrition claims can exceed 100%. Nutrition claims include any nutrient content claims, health claims, and/or front-of-pack symbols (Supplementary Table S1 for details).

**Table 2:** Proportion of foods and beverages carrying nutrient content claims that would or would not be eligible to carry such claims according to the FSANZ-NPSC, by Schedule M food category (*n* = 15,184).

Schedule M Food Category	All Products		Nutrient Content Claims (Present)					Nutrient Content Claims (Absent)				
	*n*	*%*	*n*	*Eligible*		*Not Eligible*		*n*	*Eligible*		*Not Eligible*	
				*n*	*%*	*n*	*%*		*n*	*%*	*n*	*%*
Bakery Products	2083	13.7%	896	364	40.6%	532	59.4%	1187	210	17.7%	977	82.3%
Beverages	481	3.2%	175	138	78.9%	37	21.1%	306	79	25.8%	227	74.2%
Cereals and Other Grain Products	981	6.5%	533	400	75.0%	133	25.0%	448	398	88.8%	50	11.2%
Dairy Products and Substitutes	1237	8.1%	764	527	69.0%	237	31.0%	473	144	30.4%	329	69.6%
Desserts	827	5.4%	348	147	42.2%	201	57.8%	479	46	9.6%	433	90.4%
Dessert Toppings and Fillings	115	0.8%	20	6	30.0%	14	70.0%	95	6	6.3%	89	93.7%
Egg and Egg Substitutes	56	0.4%	27	27	100.0%	0	0.0%	29	26	89.7%	3	10.3%
Fats and Oils	535	3.5%	272	85	31.3%	187	68.8%	263	106	40.3%	157	59.7%
Marine and Fresh Water Animals	440	2.9%	197	156	79.2%	41	20.8%	243	184	75.7%	59	24.3%
Fruit and Fruit Juices	1088	7.2%	714	514	72.0%	200	28.0%	374	220	58.8%	154	41.2%
Legumes	180	1.2%	96	95	99.0%	1	1.0%	84	84	100.0%	0	0.0%
Meat, Poultry, Their Products and Substitutes	895	5.9%	308	156	50.6%	152	49.4%	587	93	15.8%	494	84.2%
Miscellaneous category	449	3.0%	132	24	18.2%	108	81.8%	317	50	15.8%	267	84.2%
Combination Dishes	1348	8.9%	459	333	72.5%	126	27.5%	889	478	53.8%	411	46.2%
Nuts and Seeds	220	1.4%	116	82	70.7%	34	29.3%	104	85	81.7%	19	18.3%
Potatoes, Sweet Potatoes and Yams	140	0.9%	73	66	90.4%	7	9.6%	67	36	53.7%	31	46.3%
Salads	70	0.5%	26	23	88.5%	3	11.5%	44	30	68.2%	14	31.8%
Sauces, Dips, Gravies and Condiments	1223	8.1%	228	99	43.4%	129	56.6%	995	268	26.9%	727	73.1%
Snacks	794	5.2%	449	147	32.7%	302	67.3%	345	77	22.3%	268	77.7%
Soups	455	3.0%	256	162	63.3%	94	36.7%	199	69	34.7%	130	65.3%
Sugars and Sweets	739	4.9%	127	23	18.1%	104	81.9%	612	4	0.7%	608	99.3%
Vegetables	828	5.5%	285	240	84.2%	45	15.8%	543	346	63.7%	197	36.3%
Total	15,184	100.0%	6501	3814	58.7%	2687	41.3%	8683	3039	35.0%	5644	65.0%

A product was considered not eligible to carry claims if it did not meet its corresponding threshold of the Food Standards Australia New Zealand Nutrient Profiling Scoring Criterion (FSANZ-NPSC). Nutrient content claims were classified according to Canadian regulations (sections B.01.503 to B.01.513 of the Food and Drug Regulations) (Supplementary Table S1 for details). Values per 100 g/mL “as purchased” were used to determine the score. A product (i.e., food or beverage) can carry more than one type of claim and thus the addition of the proportion of nutrition claims can exceed 100%.

**Table 3:** Proportion of foods and beverages carrying disease risk reduction claims that would or would not be eligible to carry such claims according to the FSANZ-NPSC, by Schedule M food category (*n* = 15,184).

Schedule M Food Category	All Products		Disease Risk Reduction Claims (Present)					Disease Risk Reduction Claims (Absent)				
	*n*	*%*	*n*	*Eligible*		*Not Eligible*		*n*	*Eligible*		*Not Eligible*	
				*n*	*%*	*n*	*%*		*n*	*%*	*n*	*%*
Bakery Products	2083	13.7%	23	14	60.9%	9	39.1%	2060	560	27.2%	1500	72.8%
Beverages	481	3.2%	n/a	n/a	n/a	n/a	n/a	481	217	45.1%	264	54.9%
Cereals and Other Grain Products	981	6.5%	82	53	64.6%	29	35.4%	899	745	82.9%	154	17.1%
Dairy Products and Substitutes	1237	8.1%	5	4	80.0%	1	20.0%	1232	667	54.1%	565	45.9%
Desserts	827	5.4%	2	2	100.0%	0	0.0%	825	191	23.2%	634	76.8%
Dessert Toppings and Fillings	115	0.8%	n/a	n/a	n/a	n/a	n/a	115	12	10.4%	103	89.6%
Egg and Egg Substitutes	56	0.4%	n/a	n/a	n/a	n/a	n/a	56	53	94.6%	3	5.4%
Fats and Oils	535	3.5%	20	20	100.0%	0	0.0%	515	171	33.2%	344	66.8%
Marine and Fresh Water Animals	440	2.9%	2	2	100.0%	0	0.0%	438	338	77.2%	100	22.8%
Fruit and Fruit Juices	1088	7.2%	44	37	84.1%	7	15.9%	1044	697	66.8%	347	33.2%
Legumes	180	1.2%	5	5	100.0%	0	0.0%	175	174	99.4%	1	0.6%
Meat, Poultry, Their Products and Substitutes	895	5.9%	1	1	100.0%	0	0.0%	894	248	27.7%	646	72.3%
Miscellaneous category	449	3.0%	n/a	n/a	n/a	n/a	n/a	449	74	16.5%	375	83.5%
Combination Dishes	1348	8.9%	1	1	100.0%	0	0.0%	1347	810	60.1%	537	39.9%
Nuts and Seeds	220	1.4%	n/a	n/a	n/a	n/a	n/a	220	167	75.9%	53	24.1%
Potatoes, Sweet Potatoes and Yams	140	0.9%	n/a	n/a	n/a	n/a	n/a	140	102	72.9%	38	27.1%
Salads	70	0.5%	n/a	n/a	n/a	n/a	n/a	70	53	75.7%	17	24.3%
Sauces, Dips, Gravies and Condiments	1223	8.1%	n/a	n/a	n/a	n/a	n/a	1223	367	30.0%	856	70.0%
Snacks	794	5.2%	n/a	n/a	n/a	n/a	n/a	794	224	28.2%	570	71.8%
Soups	455	3.0%	13	12	92.3%	1	7.7%	442	219	49.5%	223	50.5%
Sugars and Sweets	739	4.9%	n/a	n/a	n/a	n/a	n/a	739	27	3.7%	712	96.3%
Vegetables	828	5.5%	28	26	92.9%	2	7.1%	800	560	70.0%	240	30.0%
Total	15,184	100.0%	226	177	78.3%	49	21.7%	14,958	6676	44.6%	8282	55.4%

A product was considered not eligible to carry claims if it did not meet its corresponding threshold of the Food Standards Australia New Zealand Nutrient Profiling Scoring Criterion (FSANZ-NPSC). Disease risk reduction claims (a subtype of health claims) were classified according to Canadian regulations (sections B.01.601 to B01.603 of the Food and Drug Regulations) (Supplementary Table S1 for details). Values per 100 g/mL “as purchased” were used to determine the score. A product (i.e., food or beverage) can carry more than one type of claim and thus the addition of the proportion of nutrition claims can exceed 100%.

**Table 4:** Proportion of foods and beverages carrying unregulated front-of-pack (FOP) symbols that would or would not be eligible to carry such claims according to the FSANZ-NPSC, by Schedule M food category (*n* = 15,184).

Schedule M Food Category	All Products		Front-of-Pack Symbols (Present)					Front-of-Pack Symbols (Absent)				
	*n*	*%*	*n*	*Eligible*		*Not Eligible*		*n*	*Eligible*		*Not Eligible*	
				*n*	*%*	*n*	*%*		*n*	*%*	*n*	*%*
Bakery Products	2083	13.7%	484	204	42.1%	280	57.9%	1599	370	23.1%	1229	76.9%
Beverages	481	3.2%	122	54	44.3%	68	55.7%	359	163	45.4%	196	54.6%
Cereals and Other Grain Products	981	6.5%	335	246	73.4%	89	26.6%	646	552	85.4%	94	14.6%
Dairy Products and Substitutes	1237	8.1%	197	145	73.6%	52	26.4%	1040	526	50.6%	514	49.4%
Desserts	827	5.4%	185	78	42.2%	107	57.8%	642	115	17.9%	527	82.1%
Dessert Toppings and Fillings	115	0.8%	5	3	60.0%	2	40.0%	110	9	8.2%	101	91.8%
Egg and Egg Substitutes	56	0.4%	25	25	100.0%	0	0.0%	31	28	90.3%	3	9.7%
Fats and Oils	535	3.5%	92	31	33.7%	61	66.3%	443	160	36.1%	283	63.9%
Marine and Fresh Water Animals	440	2.9%	51	49	96.1%	2	3.9%	389	291	74.8%	98	25.2%
Fruit and Fruit Juices	1088	7.2%	463	350	75.6%	113	24.4%	625	384	61.4%	241	38.6%
Legumes	180	1.2%	38	38	100.0%	0	0.0%	142	141	99.3%	1	0.7%
Meat, Poultry, Their Products and Substitutes	895	5.9%	116	92	79.3%	24	20.7%	779	157	20.2%	622	79.8%
Miscellaneous category	449	3.0%	21	5	23.8%	16	76.2%	428	69	16.1%	359	83.9%
Combination Dishes	1348	8.9%	287	241	84.0%	46	16.0%	1061	570	53.7%	491	46.3%
Nuts and Seeds	220	1.4%	31	24	77.4%	7	22.6%	189	143	75.7%	46	24.3%
Potatoes, Sweet Potatoes and Yams	140	0.9%	24	19	79.2%	5	20.8%	116	83	71.6%	33	28.4%
Salads	70	0.5%	9	9	100.0%	0	0.0%	61	44	72.1%	17	27.9%
Sauces, Dips, Gravies and Condiments	1223	8.1%	66	42	63.6%	24	36.4%	1157	325	28.1%	832	71.9%
Snacks	794	5.2%	177	54	30.5%	123	69.5%	617	170	27.6%	447	72.4%
Soups	455	3.0%	123	76	61.8%	47	38.2%	332	155	46.7%	177	53.3%
Sugars and Sweets	739	4.9%	40	5	12.5%	35	87.5%	699	22	3.1%	677	96.9%
Vegetables	828	5.5%	165	156	94.5%	9	5.5%	663	430	64.9%	233	35.1%
Total	15,184	100.0%	3056	1946	63.7%	1110	36.3%	12,128	4907	40.5%	7221	59.5%

A product was considered not eligible to carry claims if it did not meet its corresponding threshold of the Food Standards Australia New Zealand Nutrient Profiling Scoring Criterion (FSANZ-NPSC). Because these claims were not specifically regulated by the Government, a decision tree was developed based on the definitions used by The National Academy of Medicine in order to classify front-of-pack symbols, as described in detail in Franco-Arellano, B.; Bernstein, J.T.; Norsen, S.; Schermel, A.; L’Abbé, M.R. Assessing nutrition and other claims on food labels: a repeated cross-sectional analysis of the Canadian food supply. *BMC Nutr.* **2017**, *3*, 74 [4]. (See Supplementary Table S1 for details). Values per 100 g/mL “as purchased” were used to determine the score. A product (i.e., food or beverage) can carry more than one type of claim and thus the addition of the proportion of nutrition claims can exceed 100%.

**Table 5:** Comparison of the nutritional composition of foods and beverages with and without nutrition claims (*n* = 15,184).

Nutrient	Presence of Claim	Nutrition Claims				Nutrient Content Claims				Disease Risk Reduction Claims				Front-of-Pack Symbols			
		*n*	Mean	SD	*p*	*n*	Mean	SD	*p*	*n*	Mean	SD	*p*	*n*	Mean	SD	*p*
**Energy (Kcal per 100 g/mL)**	Claim	6990	235	179	*p* < 0.001	6501	236	180	*p* < 0.001	226	253	200	*p* = 0.954	3056	219	173	*p* < 0.001
	No Claim	8194	270	174		8683	267	174		14,958	254	177		12,128	263	177	
**Saturated Fat (g per 100 g/mL)**	Claim	6990	2.4	6.2	*p* < 0.001	6501	2.3	4.7	*p* < 0.001	226	1.3	2.5	*p* < 0.001	3056	1.8	6.9	*p* < 0.001
	No Claim	8194	4.2	6.4		8683	4.2	7.3		14,958	3.4	6.4		12,128	3.8	6.2	
**Sodium (mg per 100 g/mL)**	Claim	6990	441	1055	*p* < 0.001	6501	455	1090	*p* < 0.001	226	231	276	*p* < 0.001	3056	328	595	*p* < 0.001
	No Claim	8194	731	2170		8683	704	2112		14,958	603	1766		12,128	665	1933	
**Sugar (g per 100 g/mL)**	Claim	6990	9.7	14.3	*p* < 0.001	6501	9.5	14.2	*p* < 0.001	226	8.8	9.4	*p* < 0.001	3056	10.1	13.9	*p* < 0.001
	No Claim	8194	13.9	18.9		8683	13.9	18.7		14,958	12.0	17.2		12,128	12.4	17.8	
**Protein (g per 100 g/mL)**	Claim	6990	7.2	7.4	*p* = 0.042	6501	7.3	7.5	*p* < 0.001	226	6.1	5.4	*p* = 0.003	3056	6.6	6.8	*p* < 0.001
	No Claim	8194	7.0	7.2		8683	6.9	7.1		14,958	7.1	7.3		12,128	7.2	7.4	
**Fibre (g per 100 g/mL)**	Claim	6990	2.7	4.4	*p* < 0.001	6501	2.7	4.5	*p* < 0.001	226	5.1	6.1	*p* < 0.001	3056	3.2	4.7	*p* < 0.001
	No Claim	8194	1.9	3.4		8683	1.9	3.4		14,958	2.2	3.9		12,128	2.0	3.7	

All values are based on food and beverage (F&Bs) nutrition information in their “as purchased” form, per 100 g/mL. SD = Standard deviation. Nutrition claims include any nutrient content claims, health claims and/or front-of-pack symbols. Statistically significant difference (*p* < 0.05) was determined by Student’s *T*-test, or Mann–Whitney *U* tests when nutrients were not normally distributed.

## Supplementary Materials

The following files are available online at http://www.mdpi.com/2072-6643/10/7/832/s1. Supplementary Table S1. Simplified Canadian nutrition claims taxonomy; Supplementary Table S2. Proportion of foods and beverages with and without nutrition claims that would or would not be eligible to carry claims (as determined by the FSANZ-NPSC), per type of claim (*n* = 15,184); Supplementary Table S3. Comparison of the nutritional composition of foods and beverages with and without nutrition claims in food categories with substantial (>40%) of products carrying claims.
