# Supplementary material for: Examining the Nutritional Quality of Canadian Packaged Foods and Beverages with and without Nutrition Claims
Source: Nutrients. 2018 Jun 27;10(7):832. doi: 10.3390/nu10070832 (PMC6073495; doi:10.3390/nu10070832)
Supplement: Supplementary file 1 [file nutrients-10-00832-s001.pdf]

Supplemental Material: Examining the nutritional quality of Canadian packaged foods and beverages with and without nutrition claims

Supplementary Table 1. Simplified Canadian nutrition claims taxonomy.

**Nutrient Content Claims[1,2]**	Examples of prescribed wording
**Vitamins and minerals**	Contains vitamins, source of X vitamin(s), contains X essential nutrients, high in X vitamin(s)/mineral(s), higher in X vitamin(s)/mineral(s), reduced, free
**Total fat**	Free of fat, low in fat, reduced in fat, lower in fat, (%) fat free, no added fat
**Trans fat**	Free of trans fat, reduced in trans fat
**Fibre**	Source of fibre, high source of fibre, very high source of fibre, more fibre
**Saturated fat**	Free of saturated fat, low in saturated fat, reduced in saturated fat, lower in saturated fat
**Cholesterol**	Free of cholesterol, low in cholesterol, reduced in cholesterol, lower in cholesterol
**Sugar**	Free of sugars, reduced in sugars, lower in sugars, no added sugars
**Sodium**	Free of sodium, low in sodium, reduced in sodium, lower in sodium, no added sodium, lightly salted
**Protein**	Source of protein, excellent source of protein, more protein
**Polyunsaturated fatty acids (PUFAs)**	Source of omega-3 PUFAs, source of omega-6 PUFAs
**Energy/Calories**	Free of energy, low in energy, reduced in energy, lower in energy, source of energy, more energy, light in energy
**Lean**	Lean, extra lean
**Disease Risk Reduction Claims[1,3]**	Examples of prescribed wording
Sodium and hypertension	"A healthy diet containing foods high in potassium and low in sodium may reduce the risk of high blood pressure, a risk factor for stroke and heart disease. (Naming the food) is sodium-free."
Calcium and osteoporosis	"A healthy diet with adequate calcium and vitamin D, and regular physical activity, help to achieve strong bones and may reduce the risk of osteoporosis. (Naming the food) is a good source of calcium."
Dietary fat, saturated fat, cholesterol, trans fatty acids and coronary heart disease	"A healthy diet low in saturated and trans fats may reduce the risk of heart disease. (Naming the food) is free of saturated and trans fats."
Fruits, vegetables and cancer	"A healthy diet rich in a variety of vegetables and fruit may help reduce the risk of some types of cancer."
Plant sterols and cholesterol lowering	"Plant sterols help reduce [or help lower] cholesterol."
Oat products and cholesterol lowering	"1 cup (X g) of *Brand X* (naming the food) supplies X % of the daily amount of the fibres shown to help reduce cholesterol"
Pysllium products and cholesterol lowering	1 cup (X g) of *Brand X* (naming the food) with psyllium supplies X % of the daily amount of fibre shown to help lower cholesterol.

Unsaturated fat and cholesterol lowering	"Replacing saturated fats with polyunsaturated and monounsaturated fats from vegetable oils helps lower cholesterol. X teaspoons (X mL) of this (naming the food) contains X% less saturated fat than X teaspoons (X g) of butter"
Barley products and cholesterol lowering	125 ml (1/2 cup) of *product* supplies X% of the daily amount of the fibre shown to help lower cholesterol
**Front-of-Pack claims[4,5]**	
**Nutrient Specific Systems**	Systems with symbols that display the amount per serving of select nutrients or use symbols based on nutrient content claim criteria.
**Summary Indicator Systems**	Systems with a single symbol, icon, or score that provide summary information about the nutrient content of a product.
**Food Group/Ingredient Systems**	Systems with symbols based on the presence of a food group or food ingredient.
**Hybrid Systems**	Systems where two or more of the NSS, FGIS or SIS were displayed.
**Calorie Specific Systems**	Systems that only display calorie or energy content.

1. All approved variation in wording included.

2. Classified according to Canadian regulations (sections B.01.503 to B.01.513 of the Food and Drug Regulations)

3. Classified according to Canadian regulations (sections B.01.601 to B01.603 of the Food and Drug Regulations)

4. As original defined by The National Academy of Medicine as described in The National Academies of Sciences Engineering Medicine (formerly Institute of Medicine) "*Examination of Front-of-Package Nutrition Rating Systems and Symbols: Phase I Report*". 2010

5. Because these claims were not specifically regulated by the Government, a decision tree was developed to classify front-of-pack symbols, as described in detail in *Franco-Arellano, B., J.T. Bernstein, S. Norsen, A. Schermel, and M.R. L'Abbé, Assessing nutrition and other claims on food labels: a repeated cross-sectional analysis of the Canadian food supply. BMC Nutrition, 2017. 3(1): p. 74.*

Supplementary Table 2. Proportion of foods and beverages with and without nutrition claims that would or would not be eligible to carry claims (as determined by the FSANZ-NPSC), per type of claim (n=15,184).

Type of claim	Claim		Healthier		Less healthy		
		n	*n*	%	*n*	%	*p*
ANY NUTRITION CLAIM	Yes	6990	4060	58.1%	2930	41.9%	p<0.001
	No	8194	2793	34.1%	5401	65.9%	
ANY NUTRIENT CONTENT CLAIM	Yes	6501	3814	58.7%	2687	41.3%	p<0.001
	No	8683	3039	35.0%	5644	65.0%	
Total fat claims	Yes	1827	1068	58.5%	759	41.5%	p<0.001
	No	13357	5785	43.3%	7572	56.7%	
Saturated fat claims	Yes	893	496	55.5%	397	44.5%	p<0.001
	No	14291	6357	44.5%	7934	55.5%	
Trans fat claims	Yes	1706	756	44.3%	950	55.7%	p=0.49
	No	13478	6097	45.2%	7381	54.8%	
Cholesterol fat claims	Yes	798	447	56.0%	351	44.0%	p<0.001

	No	14386	6406	44.5%	7980	55.5%	
Omega 3&6 fatty acids claims	Yes	445	294	66.1%	151	33.9%	p<0.001
	No	14739	6559	44.5%	8180	55.5%	
Vitamins & Minerals	Yes	2270	1593	70.2%	677	29.8%	p<0.001
	No	12914	5260	40.7%	7654	59.3%	
Fibre claims	Yes	1247	966	77.5%	281	22.5%	p<0.001
	No	13937	5887	42.2%	8050	57.8%	
Sodium claims	Yes	754	547	72.5%	207	27.5%	p<0.001
	No	14430	6306	43.7%	8124	56.3%	
Sugar claims	Yes	784	602	76.8%	182	23.2%	p<0.001
	No	14400	6251	43.4%	8149	56.6%	
Energy claims	Yes	371	212	57.1%	159	42.9%	p<0.001
	No	14813	6641	44.8%	8172	55.2%	
Protein claims	Yes	426	306	71.8%	120	28.2%	p<0.001
	No	14758	6547	44.4%	8211	55.6%	
Lean claims	Yes	69	36	52.2%	33	47.8%	p=0.29
	No	15115	6817	45.1%	8298	54.9%	
ANY DISEASE RISK REDUCTION CLAIM	Yes	226	177	78.3%	49	21.7%	p<0.001
	No	14958	6676	44.6%	8282	55.4%	
ANY FRONT-OF-PACK SYMBOL	Yes	3056	1946	63.7%	1110	36.3%	p<0.001
	No	12128	4907	40.5%	7221	59.5%	
Nutrient Specific Symbols	Yes	1033	622	60.2%	411	39.8%	p<0.001
	No	14151	6231	44.0%	7920	56.0%	
Summary Indicator Symbols	Yes	723	521	72.1%	202	27.9%	p<0.001
	No	14461	6332	43.8%	8129	56.2%	
Food Group/Ingredient Symbols	Yes	726	430	59.2%	296	40.8%	p<0.001
	No	14458	6423	44.4%	8035	55.6%	
Hybrid Symbols	Yes	724	570	78.7%	154	21.3%	p<0.001
	No	14460	6283	43.5%	8177	56.5%	
Calorie Specific Symbols	Yes	491	268	54.6%	223	45.4%	p<0.001
	No	14693	6585	44.8%	8108	55.2%	

Nutrition claims (e.g., nutrient content claims, health claims and front-of-pack symbols) are described as “any representation which states, suggests or implies that a food has particular nutritional properties including, but not limited to, the energy value and to the content of protein, fat and carbohydrates, as well as the content of vitamins and minerals”. Nutrient content claims are those that “describe the amount of a nutrient in a food" (e.g., “excellent source of calcium”). Health claims are “any representation in labelling or advertising that states, suggests, or implies that a relationship exists between consumption of a food or an ingredient in the food and a person's health”, and comprises two subtypes: disease risk reduction claims (e.g. “a healthy diet rich in a variety of vegetables and fruit may help reduce the risk of some types of cancer”) and nutrient-function claims (e.g., “This product is a source of calcium. Calcium helps build strong bones and teeth”). Detailed information in sections B.01.503 to B.01.513 and B.01.601 to B01.603 of

the Canadian *Food and Drug Regulations (nutrition labelling, nutrient content claims and health claims) 2003*. The National Academy of Medicine has defined, in its report "*Examination of Front-of-Package Nutrition Rating Systems and Symbols: Phase I Report" (2010)*, FOP symbols as "systems that use nutrient criteria and symbols to indicate that a product has certain nutritional characteristics. Symbols are often placed on the principal display panel of the product, but may also be found on the side, top, or back panels or on shelf-tags". A product can carry more than one type of claim and the addition of the proportion of nutrition claims can exceed 100%. A product was considered not eligible to carry claims if it did not meet its corresponding threshold of the Food Standards Australia New Zealand Nutrient Profiling Scoring Criterion. Values per 100g/ml "as purchased" were used to determine the score. Statistically significant difference based on chi-square test ($p<0.05$).

Supplementary Table 3. Comparison of the nutritional composition of foods and beverages with and without nutrition claims in food categories with substantial number of products (>40%) carrying claims.

Nutrient	Presence of claim	Nutrition Claims				Nutrient content claims				Disease risk reduction claims				Front-of-pack symbols			
		n	Mean	SD	*p*	*n*	Mean	SD	*p*	*n*	Mean	SD	*p*	*n*	Mean	SD	*p*
**Bakery products (n=2,083)**																	
**Energy (Kcal per 100g/ml)**	Claim	1004	362	95	p<0.001	896	360	95	p<0.001	23	330	99	p=0.02	484	372	92	p=0.187
	No Claim	1079	391	89		1187	390	89		2060	378	93		1599	379	93	
**Saturated Fat (g per 100g/ml)**	Claim	1004	2.5	3.1	p<0.001	896	2.3	3.0	p<0.001	23	1.6	1.3	p=0.004	484	2.5	2.6	p<0.001
	No Claim	1079	6.1	5.1		1187	5.9	5.1		2060	4.4	4.7		1599	4.9	5.0	
**Sodium (mg per 100g/ml)**	Claim	1004	468	281	p<0.001	896	476	288	p<0.001	23	373	147	p=0.534	484	480	277	p<0.001
	No Claim	1079	397	264		1187	397	259		2060	432	276		1599	416	272	
**Sugar (g per 100g/ml)**	Claim	1004	12.2	12.7	p<0.001	896	11.4	12.5	p<0.001	23	12.5	10.5	p=0.235	484	12.7	12.2	p<0.001
	No Claim	1079	21.4	15.2		1187	21.2	15.0		2060	17.0	14.8		1599	18.3	15.3	
**Protein (g per 100g/ml)**	Claim	1004	7.9	3.3	p<0.001	896	7.9	3.2	p<0.001	373	9.0	2.3	p<0.001	484	8.2	3.1	p<0.001
	No Claim	1079	6.3	3.1		1187	6.4	3.2		432	7.0	3.3		1599	6.7	3.2	
**Fibre (g per 100g/ml)**	Claim	1004	4.8	3.8	p<0.001	896	4.7	3.9	p<0.001	23	6.6	2.2	p<0.001	484	5.7	4.0	p<0.001
	No Claim	1079	2.4	2.3		1187	2.7	2.6		2060	3.5	3.4		1599	2.9	2.8	

Supplementary Table 3. Cont.

Nutrient	Presence of claim	Nutrition Claims				Nutrient content claims				Disease risk reduction claims				Front-of-pack symbols			
		n	Mean	SD	*p*	*n*	Mean	SD	*p*	*n*	Mean	SD	*p*	*n*	Mean	SD	*p*
**Desserts (n=827)**																	

**Energy (Kcal per 100g/ml)**	Claim	384	129	81	p<0.001	348	129	85	p<0.001	2	n/a	n/a	n/a	185	124	77	p<0.001
	No Claim	443	151	75		479	149	72		825	n/a	n/a		642	146	78	
**Saturated Fat (g per 100g/ml)**	Claim	384	1.5	2.3	p<0.001	348	1.4	2.3	p<0.001	2	n/a	n/a	n/a	185	1.3	2.0	p<0.001
	No Claim	443	4.5	3.6		479	4.4	3.5		825	n/a	n/a		642	3.7	3.6	
**Sodium (mg per 100g/ml)**	Claim	384	157	399	p<0.001	348	168	417	p<0.001	2	n/a	n/a	n/a	185	189	495	p=0.09
	No Claim	443	59	54		479	59	54		825	n/a	n/a		642	80	164	
**Sugar (g per 100g/ml)**	Claim	384	17.9	18.8	p=0.487	348	18.1	19.7	p<0.001	2	n/a	n/a	n/a	185	17.1	17.4	p=0.903
	No Claim	443	15.0	8.8		479	15.1	8.5		825	n/a	n/a		642	16.1	13.4	
**Protein (g per 100g/ml)**	Claim	384	2.9	4.8	p=0.046	348	3.0	5.1	p=0.019	2	n/a	n/a	n/a	185	3.2	5.8	p=0.642
	No Claim	443	1.9	2.0		479	1.8	1.9		825	n/a	n/a		642	2.1	2.7	
**Fibre (g per 100g/ml)**	Claim	384	0.4	0.8	p=0.183	348	0.4	0.8	p=0.021	2	n/a	n/a	n/a	185	0.5	0.7	p=0.954
	No Claim	443	0.5	0.8		479	0.5	0.8		825	n/a	n/a		642	0.5	0.8	

Supplementary Table 3. Cont.

**Nutrient**	**Presence of claim**	**Nutrition Claims**				**Nutrient content claims**				**Disease risk reduction claims**				**Front-of-pack symbols**			
		n	**Mean**	**SD**	***p***	***n***	**Mean**	**SD**	***p***	***n***	**Mean**	**SD**	***p***	***n***	**Mean**	**SD**	***p***
**Desserts toppings (n=115)**																	
**Energy (Kcal per 100g/ml)**	Claim	20	267	114	p=0.009	20	267	114	p=0.009	0	n/a	n/a	n/a	5	n/a	n/a	n/a
	No Claim	95	335	109		95	335	109		115	n/a	n/a		110			
**Saturated Fat (g per 100g/ml)**	Claim	20	0.9	1.7	p=0.150	20	0.9	1.7	p=0.150	0	n/a	n/a	n/a	5	n/a	n/a	n/a
	No Claim	95	2.9	8.2		95	2.9	8.2		115	n/a	n/a		110			

**Sodium (mg per 100g/ml)**	Claim	20	146	104	p=0.903	20	146	104	p=0.903	0	n/a	n/a	n/a	5	n/a	n/a	n/a
	No Claim	95	157	216		95	157	216		115	n/a	n/a		110			
**Sugar (g per 100g/ml)**	Claim	20	39.3	18.9	p=0.036	20	39.3	18.9	p=0.036	0	n/a	n/a	n/a	5	n/a	n/a	n/a
	No Claim	95	49.1	19.6		95	49.1	19.6		115	n/a	n/a		110			
**Protein (g per 100g/ml)**	Claim	20	0.8	0.8	p=0.025	20	0.8	0.8	p=0.025	0	n/a	n/a	n/a	5	n/a	n/a	n/a
	No Claim	95	0.7	1.2		95	0.7	1.2		115	n/a	n/a		110			
**Fibre (g per 100g/ml)**	Claim	20	0.8	1.1	p=0.495	20	0.8	1.1	p=0.495	0	n/a	n/a	n/a	5	n/a	n/a	n/a
	No Claim	95	0.8	1.4		95	0.8	1.4		115	n/a	n/a		110			

Supplementary Table 3. Cont.

**Nutrient**	**Presence of claim**	**Nutrition Claims**				**Nutrient content claims**				**Disease risk reduction claims**				**Front-of-pack symbols**			
		n	**Mean**	**SD**	***p***	***n***	**Mean**	**SD**	***p***	***n***	**Mean**	**SD**	***p***	***n***	**Mean**	**SD**	***p***
**Fats and Oils (n=535)**																	
**Energy (Kcal per 100g/ml)**	Claim	277	450	261	p<0.001	272	451	260	p<0.001	20	n/a	n/a	n/a	92	384	263	p<0.001
	No Claim	258	584	235		263	580	238		515	n/a	n/a		443	542	248	
**Saturated Fat (g per 100g/ml)**	Claim	277	7.8	10.4	p<0.001	272	7.8	10.4	p<0.001	20	n/a	n/a	n/a	92	4.8	4.3	p<0.001
	No Claim	258	11.9	13.9		263	11.7	13.9		515	n/a	n/a		443	10.8	13.2	
**Sodium (mg per 100g/ml)**	Claim	277	657	606	p=0.003	272	651	599	p=0.006	20	n/a	n/a	n/a	92	705	385	p=0.01
	No Claim	258	489	493		263	498	507		515	n/a	n/a		443	549	587	
**Sugar (g per 100g/ml)**	Claim	277	5.7	9.3	p=0.288	272	5.6	9.4	p=0.363	20	n/a	n/a	n/a	92	5.7	7.5	p=0.187
	No Claim	258	4.4	7.1		263	4.5	7.1		515	n/a	n/a		443	5.0	8.5	

**Protein (g per 100g/ml)**	Claim	277	0.7	1.0	p=0.016	272	0.7	1.0	p=0.018	20	n/a	n/a	n/a	92	0.6	0.7	p=0.811
	No Claim	258	0.5	0.8		263	0.5	0.8		515	n/a	n/a		443	0.6	0.9	
**Fibre (g per 100g/ml)**	Claim	277	0.0	0.4	p=0.171	272	0.0	0.5	p=0.163	20	n/a	n/a	n/a	92	0.1	0.7	p=0.217
	No Claim	258	0.0	0.0		263	0.0	0.0		515	n/a	n/a		443	0.0	0.2	

Supplementary Table 3. Cont.

**Nutrient**	**Presence of claim**	**Nutrition Claims**				**Nutrient content claims**				**Disease risk reduction claims**				**Front-of-pack symbols**			
		n	**Mean**	**SD**	***p***	***n***	**Mean**	**SD**	***p***	***n***	**Mean**	**SD**	***p***	***n***	**Mean**	**SD**	***p***
**Meat, Poultry, and Substitutes (n=895)**																	
**Energy (Kcal per 100g/ml)**	Claim	312	183	82	p<0.001	308	183	81	p<0.001	1	n/a	n/a	n/a	116	161	62	p<0.001
	No Claim	583	235	85		587	234	85		894	n/a	n/a		779	225	87	
**Saturated Fat (g per 100g/ml)**	Claim	312	2.7	3.4	p<0.001	308	2.7	3.4	p<0.001	1	n/a	n/a	n/a	116	1.8	2.4	p<0.001
	No Claim	583	5.7	3.9		587	5.7	3.9		894	n/a	n/a		779	5.1	4.0	
**Sodium (mg per 100g/ml)**	Claim	312	676	420	p<0.001	308	673	420	p<0.001	1	n/a	n/a	n/a	116	508	367	p<0.001
	No Claim	583	819	465		587	820	464		894	n/a	n/a		779	808	454	
**Sugar (g per 100g/ml)**	Claim	312	2.1	4.7	p=0.545	308	2.2	4.7	p=0.452	1	n/a	n/a	n/a	116	1.8	3.1	p=0.411
	No Claim	583	1.8	3.3		587	1.8	3.3		894	n/a	n/a		779	1.9	4.0	
**Protein (g per 100g/ml)**	Claim	312	18.3	7.5	p<0.001	308	18.3	7.5	p<0.001	1	n/a	n/a	n/a	116	17.8	5.5	p=0.001
	No Claim	583	16.5	5.2		587	16.5	5.2		894	n/a	n/a		779	17.0	6.2	
	Claim	312	0.8	1.3	p<0.001	308	0.8	1.3	p<0.001	1	n/a	n/a	n/a	116	1.1	1.5	p<0.001

Nutrient	Presence of claim	n	Mean	SD	p	n	Mean	SD	p	n	Mean	SD	p	n	Mean	SD	p
**Fibre (g per 100g/ml)**	No Claim	583	0.4	0.8		587	0.4	0.8		894	n/a	n/a		779	0.4	0.9	

Supplementary Table 3. Cont.

**Nutrient**	**Presence of claim**	**Nutrition Claims**				**Nutrient content claims**				**Disease risk reduction claims**				**Front-of-pack symbols**			
		n	**Mean**	**SD**	***p***	***n***	**Mean**	**SD**	***p***	***n***	**Mean**	**SD**	***p***	***n***	**Mean**	**SD**	***p***
**Miscellaneous category (n=449)**																	
**Energy (Kcal per 100g/ml)**	Claim	137	308	131	p=0.568	132	310	132	p=0.803	0	n/a	n/a	n/a	21	338	75	p=0.634
	No Claim	312	298	159		317	297	158		449	n/a	n/a		428	299	154	
**Saturated Fat (g per 100g/ml)**	Claim	137	5.3	29.4	p=0.511	132	1.1	1.9	p=0.833	0	n/a	n/a	n/a	21	28.4	72.2	p=0.813
	No Claim	312	1.5	2.8		317	3.3	19.6		449	n/a	n/a		428	1.4	2.6	
**Sodium (mg per 100g/ml)**	Claim	137	2669	4475	p=0.055	132	2759	4535	p=0.147	0	n/a	n/a	n/a	21	739	843	p=0.003
	No Claim	312	4726	7891		317	4656	7848		449	n/a	n/a		428	4263	7215	
**Sugar (g per 100g/ml)**	Claim	137	19.2	18.0	p=0.016	132	18.9	17.8	p=030	0	n/a	n/a	n/a	21	20.6	19.3	p=0.302
	No Claim	312	16.9	22.5		317	17.0	22.6		449	n/a	n/a		428	17.4	21.4	
**Protein (g per 100g/ml)**	Claim	137	8.8	7.1	p=0.430	132	8.9	7.1	p=0.262	0	n/a	n/a	n/a	21	7.2	4.2	p=0.890
	No Claim	312	9.3	9.6		317	9.2	9.5		449	n/a	n/a		428	9.2	9.0	
**Fibre (g per 100g/ml)**	Claim	137	4.1	5.7	p<0.001	132	4.1	5.8	p=0.001	0	n/a	n/a	n/a	21	6.4	7.6	p=0.032
	No Claim	312	2.9	6.2		317	2.9	6.2		449	n/a	n/a		428	3.1	6.0	

Supplementary Table 3. Cont.

Nutrient	Presence of claim	Nutrition Claims				Nutrient content claims				Disease risk reduction claims				Front-of-pack symbols			
		n	Mean	SD	*p*	*n*	Mean	SD	*p*	*n*	Mean	SD	*p*	*n*	Mean	SD	*p*
**Sauces, Dips, Gravies (n=1,223)**																	
**Energy (Kcal per 100g/ml)**	Claim	238	149	119	p=0.332	228	152	121	p=0.448	0	n/a	n/a	n/a	66	90	66	p<0.001
	No Claim	985	152	118		995	152	117		1223	n/a	n/a		1157	155	119	
**Saturated Fat (g per 100g/ml)**	Claim	238	1.1	1.8	p=0.004	228	1.1	1.8	p=0.010	0	n/a	n/a	n/a	66	0.8	1.2	P=0.158
	No Claim	985	1.3	2.6		995	1.3	2.6		1223	n/a	n/a		1157	1.3	2.5	
**Sodium (mg per 100g/ml)**	Claim	238	1562	2292	p=0.036	228	1612	2329	p=0.097	0	n/a	n/a	n/a	66	750	1871	p<0.001
	No Claim	985	1289	1892		995	1280	1885		1223	n/a	n/a		1157	1376	1980	
**Sugar (g per 100g/ml)**	Claim	238	9.7	12.7	p=0.136	228	9.9	13.0	p=0.225	0	n/a	n/a	n/a	66	6.1	6.8	p=0.231
	No Claim	985	12.6	15.3		995	12.5	15.2		1223	n/a	n/a		1157	12.4	15.1	
**Protein (g per 100g/ml)**	Claim	238	4.8	6.2	p<0.001	228	4.8	6.3	p<0.001	0	n/a	n/a	n/a	66	2.6	2.2	p=0.139
	No Claim	985	2.6	3.3		995	2.6	3.3		1223	n/a	n/a		1157	3.0	4.2	
**Fibre (g per 100g/ml)**	Claim	238	1.6	2.5	p=0.006	228	1.6	4.1	p=0.029	0	n/a	n/a	n/a	66	1.5	1.3	p<0.001
	No Claim	985	1.0	1.9		995	1.1	1.9		1223	n/a	n/a		1157	1.1	2.5	

Supplementary Table 3. Cont.

Nutrient	Presence of claim	Nutrition Claims				Nutrient content claims				Disease risk reduction claims				Front-of-pack symbols			
		n	Mean	SD	*p*	*n*	Mean	SD	*p*	*n*	Mean	SD	*p*	*n*	Mean	SD	*p*
**Snacks (n=794)**																	
**Energy (Kcal per 100g/ml)**	Claim	478	486	90	p<0.001	449	488	90	p<0.001	0	n/a	n/a	n/a	177	447	93	p<0.001
	No Claim	316	528	84		345	522	87		794	n/a	n/a		617	519	83	

**Saturated Fat (g per 100g/ml)**	Claim	478	3.5	3.3	p<0.001	449	3.4	3.2	p<0.001	0	n/a	n/a	n/a	177	2.8	3.4	p<0.001
	No Claim	316	5.6	3.9		345	5.6	3.9		794	n/a	n/a		617	4.8	3.6	
**Sodium (mg per 100g/ml)**	Claim	478	534	394	p=0.105	449	529	393	p=0.35	0	n/a	n/a	n/a	177	501	406	p=0.371
	No Claim	316	492	372		345	503	376		794	n/a	n/a		617	522	380	
**Sugar (g per 100g/ml)**	Claim	478	11.4	20.3	p=0.95	449	10.6	19.3	p=0.164	0	n/a	n/a	n/a	177	18.3	25.8	p<0.001
	No Claim	316	11.3	14.3		345	12.4	16.4		794	n/a	n/a		617	9.4	14.7	
**Protein (g per 100g/ml)**	Claim	478	9.0	6.7	p<0.001	449	9.2	6.8	p<0.001	0	n/a	n/a	n/a	177	8.1	6.8	p<0.001
	No Claim	316	13.1	7.5		345	12.5	7.5		794	n/a	n/a		617	11.3	7.3	
**Fibre (g per 100g/ml)**	Claim	478	5.6	3.7	p=0.525	449	5.7	3.7	p=0.515	0	n/a	n/a	n/a	177	5.9	4.8	p=0.89
	No Claim	316	5.8	3.5		345	5.6	3.5		794	n/a	n/a		617	5.6	3.2	

Supplementary Table 3. Cont.

**Nutrient**	**Presence of claim**	**Nutrition Claims**				**Nutrient content claims**				**Disease risk reduction claims**				**Front-of-pack symbols**			
		n	**Mean**	**SD**	***p***	***n***	**Mean**	**SD**	***p***	***n***	**Mean**	**SD**	***p***	***n***	**Mean**	**SD**	***p***
**Sugars and Sweets (n=739)**																	
**Energy (Kcal per 100g/ml)**	Claim	147	300	153	p<0.001	127	284	151	p<0.001	0	n/a	n/a	n/a	40	332	158	p=0.08
	No Claim	592	395	136		612	395	136		739	n/a	n/a		699	378	144	
**Saturated Fat (g per 100g/ml)**	Claim	147	5.0	7.8	p<0.001	127	4.1	7.7	p<0.001	0	n/a	n/a	n/a	40	6.3	7.0	p=0.61
	No Claim	592	8.3	9.6		612	8.4	9.6		739	n/a	n/a		699	7.7	9.5	
**Sodium (mg per 100g/ml)**	Claim	147	106	317	p=0.002	127	108	339	p=0.008	0	n/a	n/a	n/a	40	72	106	p=0.69
	No Claim	592	57	79		612	58	80		739	n/a	n/a		699	66	161	

**Sugar (g per 100g/ml)**	Claim	147	38.9	22.0	$p<0.001$	127	38.2	22.2	$p<0.001$	0	n/a	n/a	n/a	40	43.5	20.8	$p=0.02$
	No Claim	592	53.3	14.6		612	53.0	14.9		739	n/a	n/a		699	50.8	17.0	
**Protein (g per 100g/ml)**	Claim	147	2.8	3.2	$p=0.54$	127	2.6	3.1	$p=0.28$	0	n/a	n/a	n/a	40	2.5	3.1	$p=0.30$
	No Claim	592	3.4	3.6		612	3.5	3.6		739	n/a	n/a		699	3.4	3.6	
**Fibre (g per 100g/ml)**	Claim	147	2.0	4.6	$p=0.25$	127	1.5	3.1	$p=0.034$	0	n/a	n/a	n/a	40	3.1	6.9	$p=0.55$
	No Claim	592	1.5	2.7		612	1.7	3.2		739	n/a	n/a		699	1.6	2.8	

All values are based on F&B nutrition information in their “as purchased” form, per 100g/ml. SD= Standard deviation. Nutrition claims include any nutrient content claims, health claims and/or front-of-pack symbols. Statistically significant difference ($p<0.05$) was determined by Student’s T-test or Mann–Whitney U tests (for those nutrients that were not normally distributed). N/A = Analyses could not be performed given the limited number of products in one group.
